# Sodium Bicarbonate Ingestion Improves Time-to-Exhaustion Cycling Performance and Alters Estimated Energy System Contribution: A Dose-Response Investigation

**DOI:** 10.3389/fnut.2020.00154

**Published:** 2020-09-08

**Authors:** William H. Gurton, Lewis A. Gough, S. Andy Sparks, Mark A. Faghy, Katharine E. Reed

**Affiliations:** ^1^School of Sport, Rehabilitation and Exercise Science, University of Essex, Colchester, United Kingdom; ^2^Research Centre for Life and Sport Sciences (CLaSS) School of Health Sciences, Birmingham City University, Birmingham, United Kingdom; ^3^Sports Nutrition and Performance Group, Department of Sport and Physical Activity, Edge Hill University, Ormskirk, United Kingdom; ^4^Human Sciences Research Centre, University of Derby, Derby, United Kingdom

**Keywords:** anaerobic, ergogenic aid, high-intensity exercise, alkalosis, fatigue, extracellular buffer

## Abstract

This study investigated the effects of two sodium bicarbonate (NaHCO_3_) doses on estimated energy system contribution and performance during an intermittent high-intensity cycling test (HICT), and time-to-exhaustion (TTE) exercise. Twelve healthy males (stature: 1.75 ± 0.08 m; body mass: 67.5 ± 6.3 kg; age: 21.0 ± 1.4 years; maximal oxygen consumption: 45.1 ± 7.0 ml.kg.min^−1^) attended four separate laboratory visits. Maximal aerobic power (MAP) was identified from an incremental exercise test. During the three experimental visits, participants ingested either 0.2 g.kg^−1^ BM NaHCO_3_ (SBC2), 0.3 g.kg^−1^ BM NaHCO_3_ (SBC3), or 0.07 g.kg^−1^ BM sodium chloride (placebo; PLA) at 60 min pre-exercise. The HICT involved 3 × 60 s cycling bouts (90, 95, 100% MAP) interspersed with 90 s recovery, followed by TTE cycling at 105% MAP. Blood lactate was measured after each cycling bout to calculate estimates for glycolytic contribution to exercise. Gastrointestinal (GI) upset was quantified at baseline, 30 and 60 min post-ingestion, and 5 min post-exercise. Cycling TTE increased for SBC2 (+20.2 s; *p* = 0.045) and SBC3 (+31.9 s; *p* = 0.004) compared to PLA. Glycolytic contribution increased, albeit non-significantly, during the TTE protocol for SBC2 (+7.77 kJ; *p* = 0.10) and SBC3 (+7.95 kJ; *p* = 0.07) compared to PLA. GI upset was exacerbated post-exercise after SBC3 for nausea compared to SBC2 and PLA (*p* < 0.05), whilst SBC2 was not significantly different to PLA for any symptom (*p* > 0.05). Both NaHCO_3_ doses enhanced cycling performance and glycolytic contribution, however, higher doses may maximize ergogenic benefits.

## Introduction

High-intensity interval training (HIIT) involves near maximal exercise bouts (>80–100% maximum heart rate) separated by brief recovery periods (1). The high anaerobic demand associated with maximal efforts results in the accumulation of hydrogen cations (H^+^) within the cytosol (2). Whilst these are mostly removed by intramuscular and/or extracellular buffering mechanics, production overwhelms neutralization, and this contributes toward a reduced intramuscular pH (3), causing exercise-induced acidosis. Such a biochemical state has been suggested to reduce glycolytic energy production and may disrupt calcium ion cross-bridge formation (4). A common strategy to mitigate these deleterious effects of exercise is to enhance circulating level of extracellular blood bicarbonate (${\text{HCO}}_{3^{-}}$), which subsequently allows for sustained efflux of H^+^ from intramuscular environments during high-intensity exercise (5). Increases in [${\text{HCO}}_{3^{-}}$] of ~5.0–6.0 mmol.l^−1^ are suggested to be ergogenic and can be achieved via the ingestion of extracellular buffers, such as sodium bicarbonate (NaHCO_3_) in doses of 0.2–0.3 g.kg^−1^ BM, respectively (6, 7).

Common practice is to ingest 0.3 g.kg^−1^ BM NaHCO_3_ at 60–90 min prior to exercise, which is based on historical research showing time to peak pH or ${\text{HCO}}_{3^{-}}$ occurs at this time point at the group mean level (6, 8). It is, however, likely that through following this strategy the dissociation of NaHCO_3_ within stomach acid will cause gastrointestinal (GI) upset (9), which may impair performance or dissuade athletes from using NaHCO_3_ (10, 11). Whilst, some authors have observed ergogenic benefits despite moderate GI upset (12, 13), in some cases the upset has been severe or the participant has not been able to continue with the study procedures (14, 15). The administration of smaller NaHCO_3_ doses (0.2 g.kg^−1^ BM) might therefore be preferable, as it can mitigate GI upset and also reduce the sodium load per dose which might alleviate the health risks of ingesting this supplement; although these risks are more associated with long term use of NaHCO_3_ (12, 16). McNaughton (17) reported exacerbated GI upset following higher NaHCO_3_ doses, while Gough et al. (12) observed reduced occurrence of bowel urgency and bloating for 0.2 g.kg^−1^ compared to 0.3 g.kg^−1^ BM NaHCO_3_. Reducing the dose is a simple strategy that might remove some of the negative connotations of ingesting this supplement, whilst it is far more cost effective than some of the recent strategies employed to reduce the GI upset following NaHCO_3_ ingestion, such as in enteric-coated capsules (18, 19).

Contemporary research has administered NaHCO_3_ using an individualized time-to-peak pH or ${\text{HCO}}_{3^{-}}$ approach, which is in response to studies showing that time-to-peak pH or ${\text{HCO}}_{3^{-}}$ can vary between 10 and 180 min within individuals, regardless of the ingestion method (i.e., capsule vs. fluid) (7, 12–14). In using the individual time-to-peak approach, this ensures that peak [${\text{HCO}}_{3^{-}}$] is achieved immediately before exercise, which does seem to lead to a more consistent ergogenic response (12, 14). The identification of this time-to-peak ${\text{HCO}}_{3^{-}}$ response presents a logistical challenge to athletes however, as the financial cost is high and requires specialist equipment and staff. It is plausible to suggest further research is therefore required to simplify this strategy, and to assess whether ergogenic benefits still exist for smaller NaHCO_3_ doses following administration at a standardized time point. This, in turn, could increase the practical application of this supplement, whilst also potentially limiting GI upset.

The ergogenic benefits associated with NaHCO_3_ ingestion are somewhat related to the increased activation of glycolytic energy pathways (20, 21). Whilst this is debated (22), NaHCO_3_ ingestion attenuates muscle acidosis during exercise thus preventing the allosteric inhibition of glycogen phosphorlyase and phosphofructokinase (5). This has been shown to increase estimated glycolytic contribution during HIIT protocols (20), while there is robust evidence suggesting enhanced glycolytic flux within the muscle (23). Strategies that elevate glycolytic energy system contribution may enhance exercise capacity during HIIT, however, research is yet to determine whether smaller NaHCO_3_ doses elicit a similar physiological response.

The purpose of this study therefore was to investigate the effect of 0.2 and 0.3 g.kg^−1^ BM NaHCO_3_ ingested at 60 min pre-exercise on estimated energy contribution during a high-intensity, interval cycling test (HICT), and time-to-exhaustion (TTE) cycling performance.

## Materials and Methods

### Experimental Approach to the Problem

A block randomized, across subjects counterbalanced, single-blind, placebo-controlled, crossover experimental design was implemented for this study. Participants visited the laboratory on four separate occasions to complete an incremental exercise test, familiarization, and three experimental trials. All testing was conducted at the same time of day (± 2 h) to minimize the confounding effects of circadian rhythms on exercise performance (24). Participants arrived at the laboratory in a 3-h post-prandial state, having refrained from alcohol ingestion and vigorous exercise for 24 h prior. Maximal aerobic power (MAP) was determined from the incremental exercise test and used to prescribe the exercise intensities for the HICT and TTE cycling protocols (described below). Participants completed these exercise procedures for three experimental treatment arms: (a) 0.2 g.kg^−1^ BM NaHCO_3_ (SBC2), (b) 0.3 g.kg^−1^ BM NaHCO_3_ (SBC3), or (c) 0.07 g.kg^−1^ BM sodium chloride to ensure taste-matching (placebo; PLA) (12). Participants were instructed to maintain activity levels and dietary intake throughout the study, which were assessed via written logs. All experimental trials were separated by 7 days.

### Participants

Twelve healthy males (stature: 1.75 ± 0.08 m; body mass: 67.5 ± 6.3 kg; age: 21.0 ± 1.4 years; maximal oxygen consumption: 45.1 ± 7.0 ml.kg.min^−1^) volunteered for this study. All participants were recreationally active and completed at least 60 min of vigorous exercise per week. Participants were excluded if they had any history of hypertension (>140/80 mmHg), were currently taking any medication/sports supplements, or had ingested intra- or extracellular buffering agents within the previous 6 months. The study was approved by the institutional departmental review board. Each participant was informed of the benefits and risks of the investigation prior to signing informed consent to participate in the study. Procedures were conducted in accordance with the World Medical Association's Declaration of Helsinki.

### Procedures

On the initial visit, participants performed an incremental exercise test on a cycle ergometer (Excalibur Sport, Lode, Netherlands) to determine MAP. Gaseous exchange was collected using a breath-by-breath metabolic cart (Oxycon Pro, Jaeger, Hoechberg, Germany) to determine maximal rate of oxygen consumption (VO_2max_). To determine VO_2max_, the highest 30 s rolling average was calculated. Following a 5-min warm-up (70 W; 70–90 rev.min^−1^), increments of 20 W.min^−1^ were applied until volitional exhaustion. This was deemed as the failure to maintain cycling cadence >60 rev.min^−1^ despite verbal encouragement. Maximal anaerobic power was calculated as the fraction of time in the final stage divided by test increment, added to completed power (25). Familiarization to exercise procedures (HICT and TTE cycling) was completed after 30 min of passive recovery. This involved three bouts of 60 s cycling (90, 95, and 100% MAP), interspersed with 90 s of active recovery (100 W) and TTE cycling at 105% MAP. These were completed on the cycle ergometer, with handle bar and seat height position adjusted according to preference, which was subsequently replicated for all experimental trials. The TTE cycling protocol was terminated when cadence dropped 10 rev.min^−1^ below the preferred cadence, and when participants were unable to re-establish preferred cadence (range of selected cadence = 70–90 rev.min^−1^). Participants were encouraged to exercise until volitional exhaustion, but total exercise time was not revealed.

**Figure 1:**
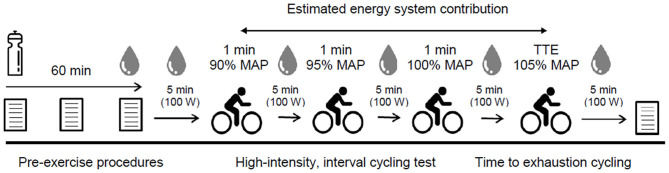
Schematic overviewing procedures during experimental visits; MAP, maximal aerobic power; TTE, time to exhaustion.

During experimental trial visits, participants completed visual analog scales (VAS) were used for baseline GI upset (0 mm = “no symptom”; 100 mm = “severest symptom”) that quantified the severity of nausea, flatulence, abdominal discomfort (AD), gut fullness (GF), bowel urgency rating (BUR), diarrhea, vomiting, and belching (12). Participants then consumed one of three experimental beverages (SBC2, SBC3, or PLA) across a 5-min period 60 min prior to exercise. Ingestion time was chosen in-line with previous work that showed the absorption kinetics between these doses are not significantly different up to this time point (14), and is the most practiced ingestion timing (6, 8). These were served as a chilled aqueous solution of 4 ml.kg^−1^ BM water and 1 ml.kg^−1^ BM squash (double strength orange squash, Tesco, UK) to increase the palatability and taste-match each beverage (26). A supplement belief questionnaire was completed post-ingestion to assess the efficacy of the single-blind design, and to ensure that no psychological bias regarding the impact of NaHCO_3_ ingestion was transferred onto participants (27). Symptoms of GI upset were repeated at 30- and 60-min post-ingestion. Pre-exercise capillary blood samples were collected into 20 μL end-to-end sodium heparised capillary tubes (EKF Diagnostic GmbH, Germany) and analyzed for blood lactate concentration ([BLa^−^]) using the Biosen C-Line (EKF Diagnostic GmbH, Germany). Participants rested for 5 min to determine baseline oxygen consumption and respiratory exchange ratio (RER), before completing the HICT and TTE protocols, during which gaseous exchange was measured throughout, and blood samples were taken after each cycling bout. Additional visual analog scales were completed immediately post-exercise for GI upset. An overview of experimental trials is displayed in Figure 1.

### Estimated Energy System Contribution Calculations

Absolute energy demand and energy contribution from the oxidative and glycolytic energetic systems were estimated via non-invasive technique. The oxidative phosphorylation pathway (W_AER_) was determined by subtracting resting oxygen consumption (i.e., the mean VO_2_ value during the final 30 s of baseline) from the area under the oxygen consumption curve for each of the three 60 s bouts (90, 95, and 100% MAP) during the HICT (28). Area under the curve was calculated using the trapezoidal method. This approach has recently been shown to provide reliable and valid estimations for W_AER_ during intermittent exercise (20, 29). The glycolytic pathway (W_[LA]_) was calculated from the assumption that a difference of 1 mmol.l^−1^ of BLa^−^ obtained by subtracting baseline [BLa^−^] from peak [BLa^−^] (i.e., delta [BLa^−^]) corresponded to 3 ml.kg^−1^ BM of O_2_ (20, 29–32). Therefore, delta [BLa^−^] for each of the three 60 s bouts and during TTE cycling (i.e., difference from pre to post) was multiplied by 3 and the participants' body mass to calculate W_[LA]_. The caloric quotient of 20.92 kJ was used to convert between absolute energy demand (in L of O_2_) and energy contribution (in kJ) for both energetic systems.

### Statistical Analysis

Normality and sphericity were assessed using Shapiro-Wilk and Mauchly tests, before correcting for any violations (Greenhouse Geisser). One-way repeated measures analysis of variance (ANOVA) were conducted for cycling TTE performance and total energy demand and contribution from W_AER_ and W_[LA]_ during exercise protocols. The smallest worthwhile change (SWC) in performance (9.1 s) was calculated as 0.3 x the between-individual SD for cycling TTE during familiarization (33). This was then used as a threshold for interpreting individual differences and in an attempt to identify a true change in exercise performance between the NaHCO_3_ and the placebo conditions. Two-factor (treatment x time) repeated measures ANOVA's were performed for [BLa^−^], RER, W_AER_, and W_[LA]_ for each of the three 60 s bouts during the HICT. When significant interactions were observed, pairwise comparisons using the bonferroni correction factor were performed. Friedman's two-way ANOVA's were conducted for GI upset. *Post-hoc* Wilcoxon matched-pair signed rank tests were performed when significance was observed, with median, *Z* score, and significance reported. Fisher's exact test was used to assess the efficacy of the single-blind design. For ANOVA interactions, effect sizes were presented as partial eta-squared ($\eta_{p}^{2}$) (34). Between treatment effect sizes were calculated by dividing the difference in means by the pooled SD (35), before applying a Hedges g (*g*) bias correction to account for the small sample size (36). These were interpreted as trivial (<0.20), small (0.20–0.49), moderate (0.50–0.79), or large (≥0.80) (37). Data are presented as mean ± SD and 95% confidence intervals (CI) reported for mean differences. Statistical significance was set at *p* < 0.05 and data were analyzed using SPSS v25 (SPSS Inc., IBM, USA).

## Results

**Figure 2:**
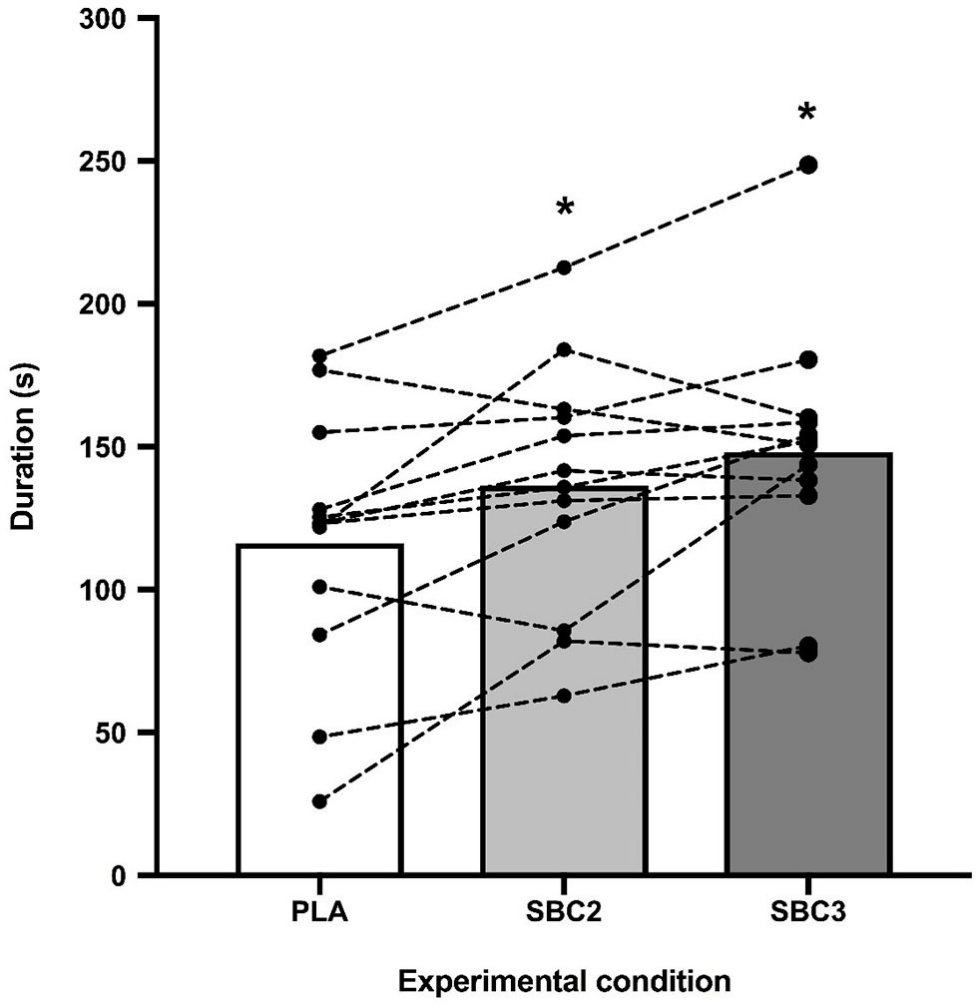
Mean differences and inter-individual variation for TTE cycling performance; SBC2, 0.2 g.kg^−1^ BM NaHCO_3_; SBC3, 0.3 g.kg^−1^ BM NaHCO_3_; PLA, sodium chloride (placebo); * sig difference compared to PLA trial (*p* < 0.05).

**Figure 3:**
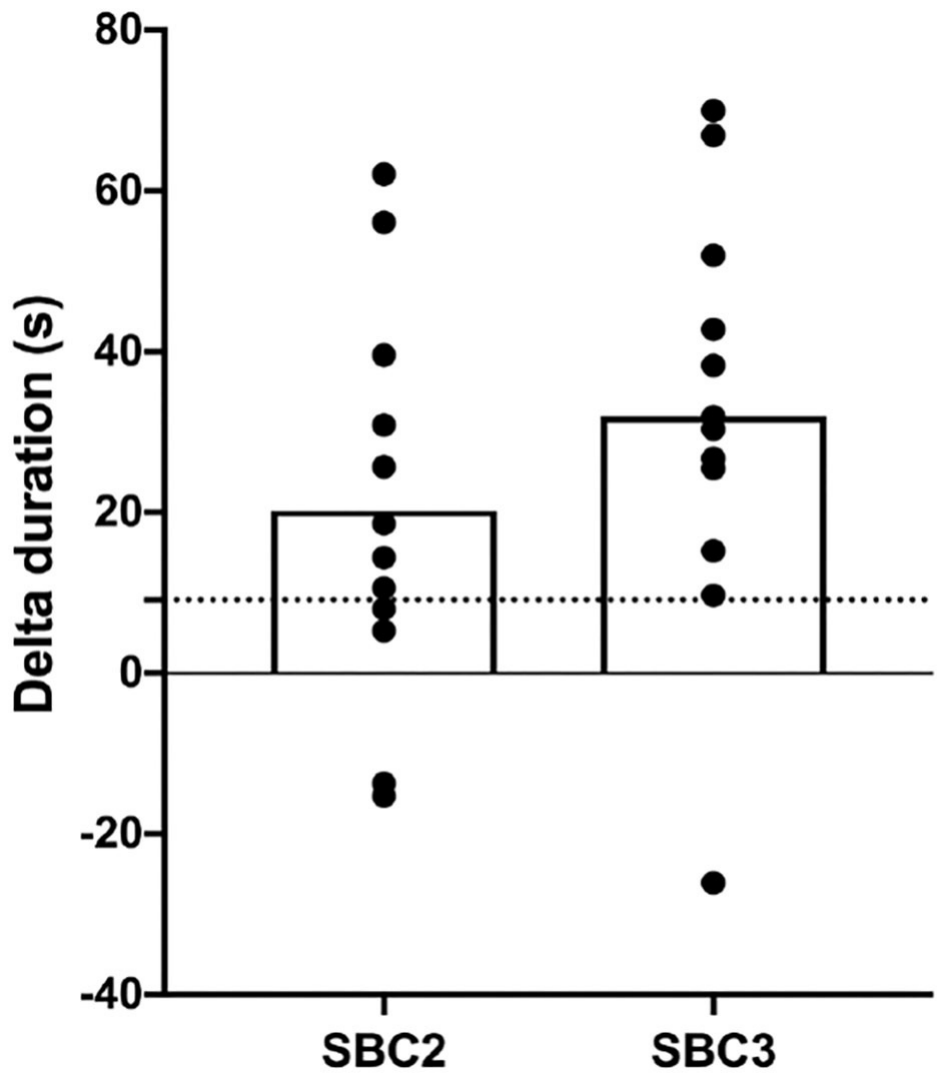
Individual changes (with mean; clear bar) in TTE duration compared to PLA condition; SBC2, 0.2 g.kg^−1^ BM NaHCO_3_; SBC3, 0.3 g.kg^−1^ BM NaHCO_3_; PLA, sodium chloride (placebo); dashed horizontal line depicts SWC in performance (9.1 s).

Performance was greater for SBC2 (136.4 ± 43.5 s) and SBC3 (158.7 ± 63.3 s) compared to PLA (116.2 ± 46.6 s) (Figure 2). These increases were significant for SBC2 (+20.2 s; CI: 0.4, 39.9; *p* = 0.045; *g* = 0.77) and SBC3 (+31.9 s; CI: 10.8, 53.1; *p* = 0.004; *g* = 1.13). A total of 8 out of 12 participants improved their performance above the SWC following SBC2, whilst 11 participants (out of 12) improved above this threshold following SBC3 (Figure 3). There was an 11.7 s mean difference in favor of SBC3 vs. SBC2, but this increase was not significant (*p* = 0.303; *g* = 0.48). Nonetheless, seven of the participants (out of 12) improved their performance above the SWC for SBC3 vs. SBC2, whilst this was only in favor of SBC2 for a single participant.

Grouped mean ± SD data for [BLa^−^] and RER are presented in Table 1. No significant differences were displayed during the HICT protocol (*p* > 0.05). Post-TTE [BLa^−^] was elevated for SBC2 (+2.35 mmol.l^−1^; CI: 0.06, 4.64; *p* = 0.04; *g* = 0.77) and SBC3 (+3.13 mmol.l^−1^; CI: 1.44, 4.82; *p* = 0.001; *g* = 1.40) compared to PLA. There was a small effect size for SBC3 vs. SBC2 (+0.78 mmol.l^−1^; *p* = 0.34; *g* = 0.46). Peak RER was also increased for SBC2 (+0.09 AU; CI: 0.03, 0.15; *p* = 0.005; *g* = 1.14) and SBC3 (+0.11 AU; CI: 0.03, 0.19; *p* = 0.011; *g* = 0.98) compared to PLA.

**Table 1:** Physiological variables ([BLa^−^] and RER) obtained during the HICT and TTE cycling.

		**90%**	**95%**	**100%**	**TTE**
[BLa^−^] (mmol.l^−1^)	SBC2	4.71 ± 1.38	6.91 ± 1.52	8.73 ± 1.80	14.09 ± 3.95^*^
	SBC3	4.30 ± 1.43	6.86 ± 1.66	9.35 ± 3.68	14.87 ± 3.01^*^
	PLA	4.26 ± 1.43	6.79 ± 2.06	8.13 ± 2.46	11.74 ± 3.47
RER (AU)	SBC2	1.08 ± 0.06	1.07 ± 0.04	1.08 ± 0.03	1.25 ± 0.06^*^
	SBC3	1.11 ± 0.05	1.09 ± 0.06	1.09 ± 0.06	1.26 ± 0.10^*^
	PLA	1.08 ± 0.07	1.07 ± 0.06	1.05 ± 0.04	1.15 ± 0.06

*Data are mean ± SD; HICT, high-intensity, interval cycling test (60 s bouts at 90, 95, and 100% maximal aerobic power); TTE, time to exhaustion; SBC2, 0.2 g.kg^−1^ BM NaHCO_3_; SBC3, 0.3 g.kg^−1^ BM NaHCO_3_; PLA, sodium chloride (placebo)*.

* *sig difference compared to PLA (p < 0.05)*.

**Figure 4:**
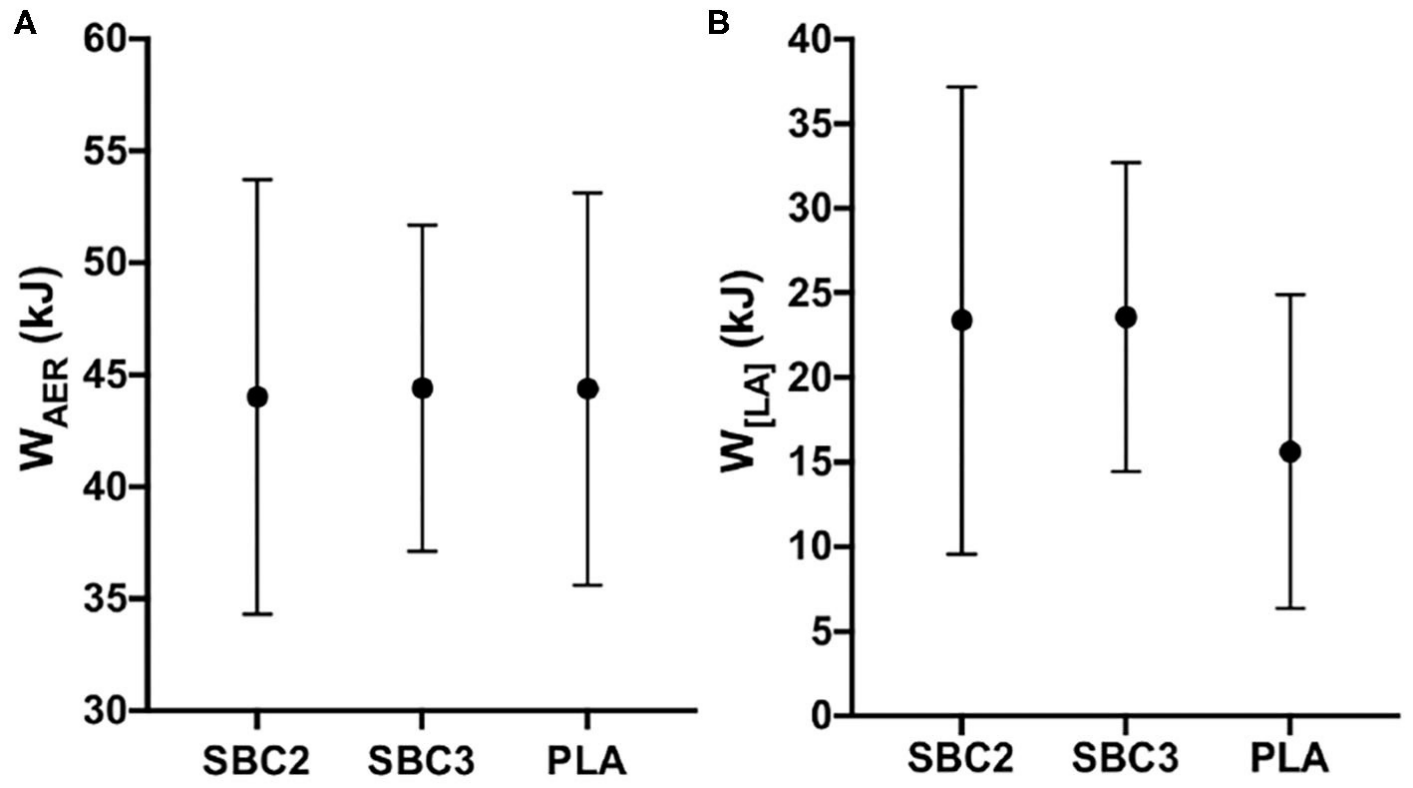
**(A,B)** Mean ± SD for W_AER_ **(A)** and W_[LA]_ **(B)** contribution during TTE cycling; SBC2, 0.2 g.kg^−1^ BM NaHCO_3_; SBC3, 0.3 g.kg^−1^ BM NaHCO_3_; PLA, sodium chloride (placebo).

Total energy demand and contribution of the oxidative and glycolytic energetic systems during the HICT are presented in Table 2. No significant differences were displayed for energy demand or contribution from W_AER_ or W_[LA]_ (*p* > 0.05), although W_[LA]_ contribution was moderately increased for SBC2 (+3.71 kJ; *p* = 0.09; *g* = 0.66) and SBC3 (+7.12 kJ; *p* = 0.14; *g* = 0.60) compared to PLA (23.40 ± 8.93 kJ). There was a small effect size for W_[LA]_ contribution when comparing SBC3 vs. SBC2 (+3.41 kJ; *p* = 0.99; *g* = 0.27). Energy contribution from W_AER_ was greater during the second 60 s bout for PLA vs. SBC2 (+4.16 kJ; CI: 0.50, 7.81; *p* = 0.03; *g* = 0.86). No significant differences were observed for energy contribution from W_AER_ or W_[LA]_ during TTE cycling (*p* > 0.05; Figures 4A,B), although W_[LA]_ was moderately increased for SBC2 (+7.77 kJ; *p* = 0.10; *g* = 0.65) and SBC3 (+7.95 kJ; *p* = 0.07; *g* = 0.70) compared to PLA (15.62 ± 9.27 kJ). No difference was reported for W_[LA]_ when comparing SBC3 vs. SBC2 (+0.18 kJ; *p* = 1.00; *g* = 0.01).

**Table 2:** Total energy demand and contribution of the oxidative and glycolytic systems during the HICT.

		**SBC2**	**SBC3**	**PLA**
Energy demand (L of O_2_)	W_AER_	5.1 ± 0.9	5.1 ± 0.8	5.3 ± 0.8
	W_[LA]_	1.3 ± 0.4	1.5 ± 0.8	1.1 ± 0.4
Energy contribution (kJ)	W_AER_	105.8 ± 18.9	106.4 ± 17.0	110.1 ± 17.2
	W_[LA]_	27.1 ± 8.5	30.5 ± 17.4	23.4 ± 8.9

*Data are mean ± SD; HICT, high-intensity, interval cycling test (60 s bouts at 90, 95, and 100% maximal aerobic power); W_AER_, oxidative phosphorylation contribution; W_[LA]_, glycolytic contribution; SBC2, 0.2 g.kg^−1^ BM NaHCO_3_; SBC3, 0.3 g.kg^−1^ BM NaHCO_3_; PLA, sodium chloride (placebo)*.

**Figure 5:**
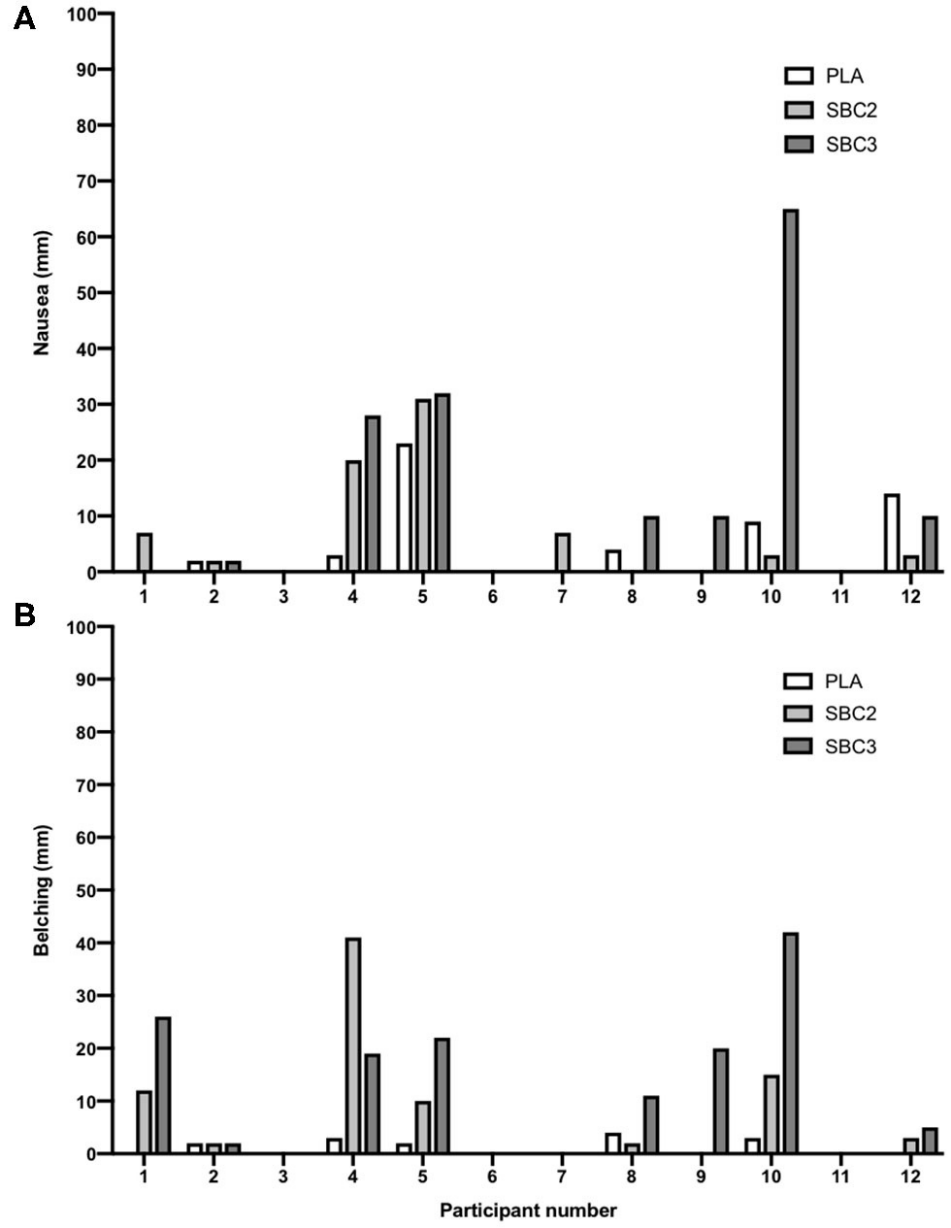
**(A,B)** Inter-individual variations in post-exercise nausea and belching; self-reported symptoms via visual analog scales (out of 100 mm); SBC2, 0.2 g.kg^−1^ BM NaHCO_3_; SBC3, 0.3 g.kg^−1^ BM NaHCO_3_; PLA, sodium chloride (placebo).

Treatments were successfully single-blinded and taste-matched (Fisher's exact test, *p* = 0.28). One subject identified all three beverages, eight only correctly perceived one of the three beverages, and the remaining three were unsure on all treatments. Eight participants reported their severest symptom after either SBC2 (4/12) or SBC3 (4/12), although some reported no difference between treatments (3/12), whereas one experienced the severest symptom following PLA (Table 3). No intervention or time interaction was observed at 30- or 60-min post-ingestion for any GI symptom (*p* > 0.05), or at post-exercise for vomiting, flatulence, GF, BUR, or diarrhea (*p* > 0.05). Nonetheless, symptom severity was increased post-exercise following SBC3 compared to PLA for nausea (10.0 vs. 1.0 mm; *Z* = −2.197; *p* = 0.028) and belching (8.0 vs. 1.0 mm; *Z* = −2.371; *p* = 0.018), but not for SBC2 compared to PLA (*p* > 0.05). Increases in the severity of nausea post-exercise was also observed following SBC3 compared to SBC2 (*Z* = 2.366; *p* = 0.018; Figure 5A), but not belching (*Z* = 1.352; *p* = 0.176; Figure 5B). There was no difference between aggregate GI upset between SBC2 and SBC3 at any time point (all *p* > 0.05).

**Table 3:** The severest gastrointestinal (GI) symptoms for participants during each experimental trial.

**Participant**	**SBC2**	**SBC3**	**PLA**
**1**	BUR (90.0)^**^	Vomiting (80.0)^**^	GF (39.0)
**2**	**BUR (19.0)**	GF (17.0)	GF (14.0)
**3**	**Belching (20.0)**	GF (18.0)	Belching (5.0)
**4**	**GF (24.0)**	**GF (24.0)**	Belching (23.0)
**5**	Nausea (31.0)	**AD (33.0)**	Nausea (23.0)
**6**	**GF (59.0)**	**GF (59.0)**	Belching (39.0)
**7**	**GF (12.0)**	GF (10.0)	Nil (0.0)
**8**	GF (39.0)	AD (31.0)	**GF (69.0)**
**9**	GF (10.0)	**Flatulence (49.0)**	Belching (21.0)
**10**	Flatulence (21.0)	**AD (71.0)**	AD (13.0)
**11**	**Nil (0.0)**	**Nil (0.0)**	**Nil (0.0)**
**12**	GF (17.0)	**GF (72.0)**	GF (66.0)

*Symptom scores (out of 100 mm) are displayed in parenthesis; SBC2, 0.2 g.kg^−1^ BM NaHCO_3_; SBC3, 0.3 g.kg^−1^ BM NaHCO_3_; PLA, sodium chloride (placebo); BUR, bowel urgency rating; GF, gut fullness; AD, abdominal discomfort*;

** *Reported 5–10 min after laboratory visit; highest symptom severity for each participant highlighted in bold*.

## Discussion

This study is the first to explore the dose-response effects of NaHCO_3_ ingestion when administered at a standardized time point on estimated energy system contribution and performance during intermittent cycling exercise. Both 0.2 and 0.3 g.kg^−1^ BM NaHCO_3_ improved cycling TTE and estimated glycolytic contribution during HICT, therefore both doses can be employed as an ergogenic strategy. Only minimal dose-dependent differences in GI upset were observed, although the smaller dose mitigated severity of post-exercise nausea and belching. The key finding of this study therefore is that 0.2 g.kg^−1^ BM of NaHCO_3_ can increase estimated glycolytic system contribution and be ergogenic for intermittent exercise performance.

Improvements in cycling TTE were observed for SBC2 and SBC3, with the moderate-to-large effect sizes reflective of previous findings employing a similar TTE protocol (26). The present study adds to previous work (17, 38), however, that ergogenic benefits can also be observed with a lower dose of NaHCO_3_. Importantly, however, more participants improved over the SWC for SBC3 vs. SBC2, and a small effect size between treatments was observed in favor of SBC3 at the group level. This contradicts findings by McKenzie et al. (38) that displayed a 4 s difference in TTE for 0.15 and 0.3 g.kg^−1^ BM NaHCO_3_, and Gough et al. (12) where only a 0.1% variation in 4-km cycling time trial performance was present for 0.2 and 0.3 g.kg^−1^ BM doses. This discrepancy could be explained by differences in administration approach (standardized time point vs. time-to-peak), or the high-degree of inter-individual variation present in acid base balance following NaHCO_3_ ingestion. Nonetheless, based on seven participants improving their performance following SBC3 vs. SBC2 (based on SWC), it is likely the athlete will secure the largest benefit from this higher dose. These dose-dependent differences in performance could also be attributed to the timing of exercise protocols. The cycling TTE protocol commenced ~75 min after NaHCO_3_ ingestion accounting for both the warm-up and HICT, however it is expected that [${\text{HCO}}_{3^{-}}$] will continue to rise until ~80 min post-ingestion for SBC3, by which point [${\text{HCO}}_{3^{-}}$] will have started to decline for SBC2 in most individuals (12, 14). Nonetheless, athletes unable to pre-determine their time-to-peak ${\text{HCO}}_{3^{-}}$ can still employ either dosing strategy of the present study to obtain performance benefits during high-intensity cycling exercise.

Moderate, albeit non-significant, increases were observed for W_[LA]_ during the HICT without altering energy demand or contribution from W_AER_, which is in agreement to findings from recent studies (20, 21, 31). Despite not achieving statistical significance, these increases were considered substantial for both SBC2 (+15.8%) and SBC3 (+30.3%) when compared to PLA, with the relatively small absolute changes in W_[LA]_ attributed to the controlled total mechanical work during the HICT (20). The most novel finding, however, was that there may be a dose-response effect of NaHCO_3_ ingestion on changes in energy system contributions, with a small effect size present for W_[LA]_ in favor of SBC3. Considering that enhanced ${\text{HCO}}_{3^{-}}$ buffering capacity is responsible for elevating glycolytic contribution, one explanation for these dose-dependent results could relate to the total amount of H^+^ that can be neutralized. Assuming that total blood volume is ~5 L and that [${\text{HCO}}_{3^{-}}$] was as small as ~1.0 mmol.l^−1^ higher for SBC3 vs. SBC2, then the higher dose could have allowed the neutralization of an extra ~5 mmoles of H^+^ (based on the 1:1 stoichiometry of ${\text{HCO}}_{3^{-}}$ and H^+^ reaction), in theory eliciting a greater up-regulation of glycolytic contribution (20). It is important to note, however, that as the current methodology only indirectly assesses glycolytic flux (i.e., from changes in [BLa^−^]), these increases in W_[LA]_ contribution may overestimate glycolytic activation, instead reflecting greater lactate efflux from working muscles (5). Nonetheless, previous research has corroborated the findings of the present study following NaHCO_3_ ingestion (23), therefore it seems plausible that both dosing strategies partially up-regulate glycolytic activation during high-intensity cycling.

The ingestion of NaHCO_3_ resulted in mild-to-moderate GI symptoms, although both doses were well-tolerated, which agrees with previous research (14). Minimal dose-dependent differences were observed for GI upset, though the reduced post-exercise nausea and belching for SBC2 agrees with Gough et al. (12) where belching was exacerbated for the higher dose. The reduced severity of GI upset from this study could be attributed to the body mass of the participants in the present study (mean = 68 ± 6 kg) compared to those that have reported greater severity of GI upset in healthy males (15) and trained rugby players (10) (90 ± 6 and 95 ± 13 kg). Relative dosing protocols were derived during early laboratory studies to normalize post-exercise base deficit (39), and therefore fail to account for physiological differences such as body mass and the total absolute NaHCO_3_ dose. Athletes with high body mass administer a greater absolute NaHCO_3_ dose despite minimal differences in gut absorption rates, particularly for the first 60 min post-ingestion (14), which most likely exacerbates GI upset. There might be an upper threshold for absolute NaHCO_3_ doses, with doses above this exacerbating GI upset. At present, 0.2 g.kg^−1^ BM NaHCO_3_ is a suitable strategy for mitigating GI upset; however, future research could examine the effect of absolute dosage on symptom severity and exercise performance.

There are methodological limitations in the present study that future research should address. Firstly, the single-blind design of this study is a limitation that is important to note. Important methodological choices were adopted, however, to mitigate any potential impact of this design. This included the standardized verbal encouragement during exercise, and the use of a supplement belief questionnaire, as per previous research (12). The findings from the latter methodological decision suggested that the supplement was blinded from the participants and therefore the single-blind design has no impact on the efficacy of NaHCO_3_ ingestion. Moreover, our inability to quantify changes in absolute demand and contribution from the ATP-PCr energetic system is a limitation. This was due to the relatively short recovery period (90 s) between each bout of the HICT that did not allow a clear EPOC curve to form and therefore, it was decided that the ATP-PCr energy contribution calculations should be excluded from our analysis. Lastly, it was not possible to measure changes in [${\text{HCO}}_{3^{-}}$] following NaHCO_3_ ingestion in the present study. Evidence suggests, however, that the ${\text{HCO}}_{3^{-}}$ response is similar for 0.2 and 0.3 g.kg^−1^ BM NaHCO_3_ doses within ~60 min, therefore participants were likely at a similar level of alkalosis irrespective of dose (12, 14). This timing of NaHCO_3_ ingestion employed in this study was selected to assess of the potential ergogenic effects for athletes unable to adopt an individualized time-to-peak ${\text{HCO}}_{3^{-}}$ approach, or access a blood gas analyser. Based on the observed ergogenic benefits for both doses vs. PLA, it should further enhance the practical application of NaHCO_3_ supplementation to the athlete with limited funding.

## Conclusion

Ingestion of 0.2 and 0.3 g.kg^−1^ BM elevated glycolytic contribution to high intensity exercise and are ergogenic strategies to improve exercise performance. It is likely that athletes will gain increased benefit from SBC3, despite the occurrence of higher GI upset. Nonetheless, some athletes may still opt for the lower dose if this displays greater tolerability, whilst still securing an ergogenic benefit. The present study also shows that the contemporary time to peak alkalosis strategy might not be required when ingested 60 min prior to exercise, however direct comparisons between these two methods of ingestion are required.

## Data Availability Statement

The raw data supporting the conclusions of this article will be made available by the authors, without undue reservation.

## Ethics Statement

The studies involving human participants were reviewed and approved by University of Essex. The patients/participants provided their written informed consent to participate in this study.

## Author Contributions

KR, WG, and LG designed the study. WG completed the data collection, whilst WG and LG completed the majority of the manuscript, MF, SS, and KR also contributed. All authors reviewed the paper and provided feedback. LG and WG completed the preparation of the manuscript.

## Conflict of Interest

The authors declare that the research was conducted in the absence of any commercial or financial relationships that could be construed as a potential conflict of interest.

## References

[1] Islam H Townsend LK Hazell TJ. Modified sprint interval training protocols. Part I Physiological responses. Appl Physiol Nutr Met. (2017) 42:339–46. 10.1139/apnm-2016-0478 28177740

[2] Allen DG Lamb GD Westerblad H. Skeletal muscle fatigue: cellular mechanisms. Physiol Rev. (2008) 88:287–332. 10.1152/physrev.00015.2007 18195089

[3] Sahlin K. Muscle energetics during explosive activities and potential effects of nutrition and training. Sports Med. (2014) 44:167–73. 10.1007/s40279-014-0256-9 25355190 PMC4213384

[4] Fitts R. The role of acidosis in fatigue: pro perspective. Med Sci Sport Exerc. (2016) 48:2335–8. 10.1249/MSS.0000000000001043 27755382

[5] Siegler JC Marshall PW Bishop D Shaw G Green S. Mechanistic insights into the efficacy of sodium bicarbonate supplementation to improve athletic performance. Sports Med. (2016) 2:41–53. 10.1186/s40798-016-0065-9 27747796 PMC5059234

[6] Carr AJ Hopkins WG Gore CJ. Effects of acute alkalosis and acidosis on performance. Sports Med. (2011) 41:801–14. 10.2165/11591440-000000000-00000 21923200

[7] Jones RL Stellingwerff T Artioli GG Saunders B Cooper S Sale C. Dose-response of sodium bicarbonate ingestion highlights individuality in time course of blood analyte responses. Int J Sport Nutr Exe. (2016) 26:445–53. 10.1123/ijsnem.2015-0286 27098290

[8] Hadzic M Eckstein ML Schugardt M. The impact of sodium bicarbonate on performance in response to exercise duration in athletes: a systematic review. J Sport Sci Med. (2019) 18:271–81. 31191097 PMC6544001

[9] Heibel AB Perim PH Oliveira LF McNaughton LR Saunders B. Time to optimize supplementation: modifying factors influencing the individual responses to extracellular buffering agents. Front Nutr. (2018) 5:35. 10.3389/fnut.2018.00035 29868599 PMC5951986

[10] Cameron SL McLay-Cooke RT Brown RC Gray AR Fairbairn KA Increased blood pH but not performance with sodium bicarbonate supplementation in elite rugby union players. Int J Sport Nutr Exe. (2010) 20:307–21. 10.1123/ijsnem.20.4.307 20739719

[11] Saunders B Sale C Harris RC Sunderland C. Sodium bicarbonate and high-intensity-cycling capacity: variability in responses. Int J Sport Physiol. (2014) 9:627–32. 10.1123/ijspp.2013-0295 24155093

[12] Gough LA Deb SK Sparks SA McNaughton LR. Sodium bicarbonate improves 4 km time trial cycling performance when individualised to time to peak blood bicarbonate in trained male cyclists. J Sports Sci. (2018) 36:1705–12. 10.1080/02640414.2017.1410875 29183257

[13] Miller P Robinson AL Sparks SA. The effects of novel ingestion of sodium bicarbonate on repeated sprint ability. J Strength Cond Res. (2016) 30:561–8. 10.1519/JSC.0000000000001126 26815179

[14] Gough LA Deb SK Sparks AS McNaughton LR. The reproducibility of blood acid base responses in male collegiate athletes following individualised doses of sodium bicarbonate: a randomised controlled crossover study. Sports Med. (2017) 47:2117–27. 10.1007/s40279-017-0699-x 28229390

[15] Kahle LE Kelly PV Eliot KA Weiss EP. Acute sodium bicarbonate loading has negligible effects on resting and exercise blood pressure but causes gastrointestinal upset. Nutr Res. (2013) 33:479–86. 10.1016/j.nutres.2013.04.009 23746564 PMC3680785

[16] Graudal NA Hubeck-Graudal T Jürgens G. Effects of low-sodium diet vs. high-sodium diet on blood pressure, renin, aldosterone, catecholamines, cholesterol, and triglyceride (cochrane review). Am J Hypertens. (2012) 25:1–15. 10.1038/ajh.2011.210 22068710

[17] McNaughton LR. Bicarbonate ingestion: effects of dosage on 60 s cycle ergometry. J Sports Sci. (1992) 10:415–23. 10.1080/02640419208729940 1331493

[18] Hilton NP Leach NK Sparks SA Gough LA Craig MM Deb SK. A novel ingestion strategy for sodium bicarbonate supplementation in a delayed-release form: a randomised crossover study in trained males. Sports Med-Open. (2019) 5:4. 10.1186/s40798-019-0177-0 30680463 PMC6346694

[19] Hilton NP Leach NK Hilton MM Sparks SA McNaughton LR. Enteric-coated sodium bicarbonate supplementation improves high-intensity cycling performance in trained cyclists. Eur J Appl Physiol. (2020) 120:1563–73. 10.1007/s00421-020-04387-5 32388584 PMC7295736

[20] da Silva RP de Oliveira LF Saunders B. Effects of β-alanine and sodium bicarbonate supplementation on the estimated energy system contribution during high-intensity intermittent exercise. Amino Acids. (2019) 51:83–96. 10.1007/s00726-018-2643-2 30182286

[21] Lopes-Silva JP Da Silva Santos JF Artioli GG. Sodium bicarbonate ingestion increases glycolytic contribution and improves performance during simulated taekwondo combat. Eur J Sports Sci. (2018) 18:431–40. 10.1080/17461391.2018.1424942 29355092

[22] Westerblad H Håkan acidosis is not a significant cause of skeletal muscle fatigue. Med Sci Sports Exerc. (2016) 48:2339–42. 10.1249/MSS.0000000000001044 27755383

[23] Hollidge-Horvat MG Parolin ML Wong D Jones NL Heigenhauser GJF. Effect of induced metabolic alkalosis on human skeletal muscle metabolism during exercise. Am J Physiol Endocrinol Metab. (2000) 278:316–29. 10.1152/ajpendo.2000.278.2.E316 10662717

[24] Reilly T. Human circadian rhythms and exercise. Crit Rev Biomed Eng. (1990) 18:165–80. 2286092

[25] Pinot J Grappe F Determination of maximal aerobic power on the field in cycling. J Sci Cycling. (2014) 3:26–31.

[26] Higgins MF James RS Price MJ. The effects of sodium bicarbonate (NaHCO_3_) ingestion on high intensity cycling capacity. J Sports Sci. (2013) 31:972–81. 10.1080/02640414.2012.758868 23323673

[27] Gough LA Deb SK Brown D Sparks SA McNaughton LR. The effects of sodium bicarbonate ingestion on cycling performance and acid base balance recovery in acute normobaric hypoxia. J Sports Sci. (2019) 37:1464–71. 10.1080/02640414.2019.1568173 30668281

[28] di Prampero PE Ferretti G. The energetics of anaerobic muscle metabolism: a reappraisal of older and recent concepts. Resp Physiol. (1999) 118:103–15. 10.1016/S0034-5687(99)00083-3 10647856

[29] Milioni F Zagatto AM Barbieri RA Andrade VL Dos Santos JW Gobatto CA. Energy systems contribution in the running-based anaerobic sprint test. Int J Sports Med. (2017) 38:226–32. 10.1055/s-0042-117722 28192833

[30] Beneke R Pollmann C Bleif I Leithäuser RM Hütler M. How anaerobic is the wingate anaerobic test for humans? Eur J Appl Physiol. (2002) 87:388–92. 10.1007/s00421-002-0622-4 12172878

[31] Brisola GM Miyagi WE da Silva HS Zagatto AM Sodium bicarbonate supplementation improved MAOD but is not correlated with 200- and 400-m running performances: a double-blind, crossover, and placebo-controlled study. Appl Physiol Nutr Metab. (2015) 40:931–7. 10.1139/apnm-2015-0036 26300016

[32] Zagatto AM Bertuzzi R Miyagi WE Padulo J Papoti M MAOD determined in a single supramaximal test: a study on the reliability and effects of supramaximal intensities. Int J Sports Med. (2016) 37:700–7. 10.1055/s-0042-104413 27176893

[33] Hopkins WG How to interpret changes in an athletic performance test. Sport Sci. (2004) 8:1–7.

[34] Olejnik S Algina J. Generalized eta and omega squared statistics: measures of effect size for some common research designs. Psychol Methods. (2003) 8:434–47. 10.1037/1082-989X.8.4.434 14664681

[35] Nakagawa S Cuthill IC. Effect size, confidence interval and statistical significance: a practical guide for biologists. Biol Rev. (2007) 82:591–605. 10.1111/j.1469-185X.2007.00027.x 17944619

[36] Lakens D. Calculating and reporting effect sizes to facilitate cumulative science: a practical primer for t-tests and ANOVAs. Front Psychol. (2013) 4:863. 10.3389/fpsyg.2013.00863 24324449 PMC3840331

[37] Cohen J Statistical Power Analysis for the Behavioral Sciences. Hillsday, NJ: Laurence Erlbaum Associates (1988).

[38] McKenzie DC Coutts KD Stirling DR Hoeben HH Kuzara G. Maximal work production following two levels of artificially induced metabolic alkalosis. J Sports Sci. (1986) 4:35–8. 10.1080/02640418608732096 3735482

[39] Singer RB Clark JK Barker ES Crosley AP Elkinton JR. The acute effects in man of rapid intravenous infusion of hypertonic sodium bicarbonate solution: I. Changes in acid-base balance and distribution of the excess buffer base. Medicine. (1955) 34:51–95. 10.1097/00005792-195502000-00003 14355369

